# Mapping the prevalence and use of questionnaires to detect the neglected sexual side effects after prostate cancer treatment: a scoping review

**DOI:** 10.1186/s13643-021-01865-5

**Published:** 2022-01-03

**Authors:** Pierre Röscher, Ronisha Sathiram, Joanne E. Milios, Jacqueline M. van Wyk

**Affiliations:** 1 Nelson R. Mandela School of Medicine, University of KwaZulu-Natal., 719 Umbilo Rd, Berea 4001 Umbilo, South Africa; 2 Greys Hospital Pietermaritzburg, and Clinical Researcher and Professional Practice Nelson R. Mandela School of Medicine, University of KwaZulu-Natal., 719 Umbilo Rd, Berea 4001 Umbilo, South Africa; 3 School of Sport Science, Exercise & Health, The University of Western Australia., Parkway Rd, 6009 Crawley, Western Australia; 4 Nelson R. Mandela School of Medicine, University of KwaZulu-Natal., 719 Umbilo Rd, Berea 4001 Umbilo, South Africa

**Keywords:** Prostate cancer, Prevalence, Questionnaire use, Neglected sexual side effects

## Abstract

**Background:**

Early prostate cancer (PCa) treatment interventions may leave men with debilitating sexual side effects, especially when not diagnosed or present at initial follow-up treatment. Men are often embarrassed to disclose their sexual dysfunction. This may lead to sexual side effects related to PCa treatment remaining untreated, adding to their burden of disability. This study was conducted to map the evidence on the prevalence of neglected sexual side effects (NSSE) after radical prostatectomy (RP) surgery or radiation treatment (RT) for PCa treatment and the reported use of questionnaires to identify such side effects.

**Methods:**

This systematic scoping review’s search strategy involved searching MEDLINE/PubMed, Science Direct and Google Scholar databases. Guided by eligibility criteria, two independent reviewers conducted title, abstract and full-text screening. Data from the included studies were extracted. The review team explored the implications of the findings in relation to the research question and aims of the study. The Mixed Method Appraisal Tool was used to appraise the quality of the included studies. This review is reported according to the Preferred Reporting Items for Systematic Reviews and Meta-Analysis guidelines.

**Results:**

Searches of the databases identified 1369 articles, with 23 eventually included for review. The prevalence of NSSE ranged between 0 and 78% in studies reporting on early PCa treatment of RP and RT patients. Orgasmic dysfunction (5–78%), penile curvature changes (10–15.9%) and penile length shortening (0–55%) similarly showed a low to moderate prevalence. Climacturia had low prevalence (4–5.2%) after RT and moderate prevalence (21–38%) after RP, whilst anejaculation had low to high prevalence (11–72%) after RT. No validated questionnaire was used to detect any NSSE after early PCa treatment. Studies mainly modified other questionnaires, and two studies used non-validated questionnaires to identify some NSSE. Participants in the included studies reported being inadequately informed about the possible sexual side effects of their treatment.

**Conclusion:**

This study showed a low to a high prevalence of NSSE in men after RP and RT for early PCa treatment. Questionnaires helped detect individual NSSEs after PCa treatment but there is currently no evidence of a valid, reliable and comprehensive questionnaire to detect the NSSE collectively.

**Scoping review registration:**

N/A

**Supplementary Information:**

The online version contains supplementary material available at 10.1186/s13643-021-01865-5.

## Background

Prostate cancer (PCa) is a major cause of disease and morbidity amongst men, and it is the second most common cancer affecting men on a global scale [1]. Early PCa or localised PCa is cancer within the prostate described as stage I or II on the tumour-node-metastasis system [2]. Early PCa treatment consists of radical prostatectomy (RP) surgery or radiation therapy (RT), either offered through external beam radiotherapy or brachytherapy. The treatment may result in side effects such as sexual dysfunction [3] and less common physical deformities such as penile length shortening and penile curvature changes (Peyronie’s disease) [4, 5]. Sexual dysfunction from PCa treatment is common regardless of whether the treatment modality included surgical or non-surgical interventions. Sexual dysfunction is reported to increase during each year of follow-up after the initial intervention of RT, and it affects an average of 50% of patients within 5 years of receiving treatment [6].

Most men generally recover from pain and incontinence after RP but sexual side effects often remain untreated, leaving them with long-lasting and debilitating sexual dysfunction [7]. Men and their partners also suffer psychologically after PCa treatment due to anxiety and depression relating to sexual dysfunction [8]. Specific conditions related to physical, sexual dysfunction are common after PCa treatment. These conditions include orgasm-associated incontinence/climacturia, urinary incontinence during sexual stimulation, altered perception of orgasm, pain with orgasm, anejaculation, penile length shortening, and penile deformity [4, 5, 7, 9]. They are collectively referred to as the “neglected sexual side effects” (NSSE), and the symptoms are reportedly prevalent in 20–93% of RP patients [7].

Only a fifth of the men who have been diagnosed with PCa will ever discuss issues related to sexual dysfunction with their health care practitioners [10]. Clinicians may be able to use the responses from a specific patient questionnaire as a starting point to discussing issues relating to the patient’s specific symptoms of sexual dysfunction. Two validated questionnaires, the Expanded Prostate Cancer Index Composite [11] and International Index of Erectile Function [12], were recommended for use in this context in 2015 [3]. Whilst the Expanded Prostate Cancer Index Composite and International Index of Erectile Function are available to stimulate the conversation around general urinary and sexual function, there is currently no validated instrument to identify the collective symptoms specific to NSSE after early PCa treatment [4, 5, 13].

Two previous systematic reviews have explored and reported on the collective prevalence and assessment of NSSE [4, 7]. It has furthermore been established that there is no validated questionnaire to screen for NSSE and no evidence on the availability of a questionnaire to inquire about symptoms relating to NSSE in patients who had undergone treatment for PCa. It was, therefore, essential to map the evidence on the prevalence and use of questionnaires relating to the neglected sexual side effects after prostate cancer treatment to improve our understanding of NSSE and highlight knowledge gaps on the role of questionnaires in the assessment of the NSSEs.

## Methodology

A protocol for this scoping review by Roscher and van Wyk [14] can be accessed at https://rdcu.be/b7i8I.

The scoping review followed the five steps described by Arksey and O’ Malley [15] that included the following;

1. Identifying the research question
2. Identifying relevant studies
3. Study selection
4. Charting the data
5. Collating, summarising and reporting on the data

Quality assessment of each of the included primary studies was to be done as described by Levac et al. [16].

### Identifying the research questions

The research was conducted to map the prevalence of NSSE and the use of a questionnaire to identify the NSSE after prostate cancer treatment. The research questions were as follows:

- What is the prevalence of the common NSSE’s following early PCa treatment through surgical interventions/RP?
- What is the prevalence of the common NSSE following early PCa treatment through non-surgical interventions/RT?
- What are the role and use of questionnaires in detecting NSSE after early PCa treatment?

### Search strategy

A literature search was conducted using the databases MEDLINE/PubMed, Science Direct and Google Scholar to search for articles matching the research questions. Boolean terms and MeSH (Medical Subject Heading) terms were employed using the keywords: Orgas* OR Pencil* OR Climacturia OR Dysorgasmia OR anejaculation OR Peyronie OR neglected AND (prostate cancer OR prostatectomy).

### Eligibility criteria

The population, concept context (PCC) framework was used to determine the eligibility of studies for inclusion. The concept of interest was to identify studies on the prevalence of NSSE and the use of questionnaires to identify NSSE in a population of men after they had received surgical and non-surgical treatment following early PCa diagnosis.

The search was conducted on articles published between 1 January 2009 and 31 December 2019 only to include the most recent evidence on the use of questionnaires to identify NSSE. Other search parameters included original studies that were available in English and related to humans. Only studies that matched our aim in their titles were selected for further processing. The review excluded literature and grey literature outside the search period, unavailable in English and unrelated to sexual dysfunction.

### Study selection

The identification of the relevant literature followed a systematic approach. The results of all three databases were combined into one Excel spreadsheet after applying the search parameters.

The primary reviewer performed the search strategy on the databases to retrieve publications and then removed all duplicates. The titles of studies were screened to determine their eligibility for inclusion. Two reviewers screened all retrieved abstracts and they were evaluated for eligibility using the inclusion criteria. Agreement between the reviewers about potentially relevant studies was reached, and the full text was obtained for screening. Two independent reviewers did the full-text screening, and a third investigator was engaged to resolve disagreements between reviewers.

### Charting the data

A data charting form was developed to extract information on each publication and organise and synthesise information about each study (Additional file 1). The data collected included details on the author(s) and date of publication, the aim and research questions, the geographical context of the study, the population, study design and the number of participants. We also extracted information on the time reported since participants started the PCa treatment, the prevalence of NSSE and the reported use of questionnaires to identify NSSE after PCa.

The data sets were organised to answer each research question. Furthermore, the data relating to the prevalence of NSSE was organised according to the two main approaches for treating PCa, those relating to surgical approaches (RP) and those following non-surgical approaches (RT).

### Quality appraisal

An electronic version of the Mixed Method Appraisal Tool (MMAT) [17] was adapted to assess the quality of the included studies. The study designs included in this scoping review were qualitative, quantitative descriptive and mixed methods studies. The specific criteria to determine the appropriateness of each included study are outlined in Additional file 2.

Two reviewers independently performed the quality assessment, and the final scores were discussed for consensus. The overall quality for each included study was calculated according to the following MMAT guidelines (score = number of criteria met/total score in each domain). One point was allocated when the study met each of the five criteria, and a total score in the form of a percentage represents the quality of the included studies (Additional file 2).

The results used the following descriptors.

- Very poor quality (20%) where minimal criteria are met
- Poor quality (40%) where less than half the criteria are not met
- Fair quality (60%) where just more than half the criteria is met
- Good quality (80%) where most of the criteria are being met
- Excellent quality (100%) all criteria are met

The overall quality of a combination of components cannot be more than its weakest component in mixed-methods studies, making the overall score equal to the lowest-scoring component [17].

### Collating, summarising and reporting on the data

The findings of this scoping review were analysed using a deductive content analysis approach, where themes were reported to answer each research question [18]. The review team discussed findings, resolved issues, and finalised findings. The review team explored the implications of the findings in how they relate to the study’s aims and further research in the field.

The collected data was organised into subgroups (Additional file 1). The findings were analysed and reported according to the research questions. The data relating to the prevalence of the NSSE was quantitative, and the data about the use of a questionnaire yielded either one of 3 results: (i) a commonly used standardised questionnaire, (ii) an informal questionnaire, or (iii) no questionnaire. In addition to the methodologies mentioned above, the PRISMA-ScR checklist [19] guided the reporting of the scoping review (Additional file 3*)*.

## Results

**Fig. 1:**
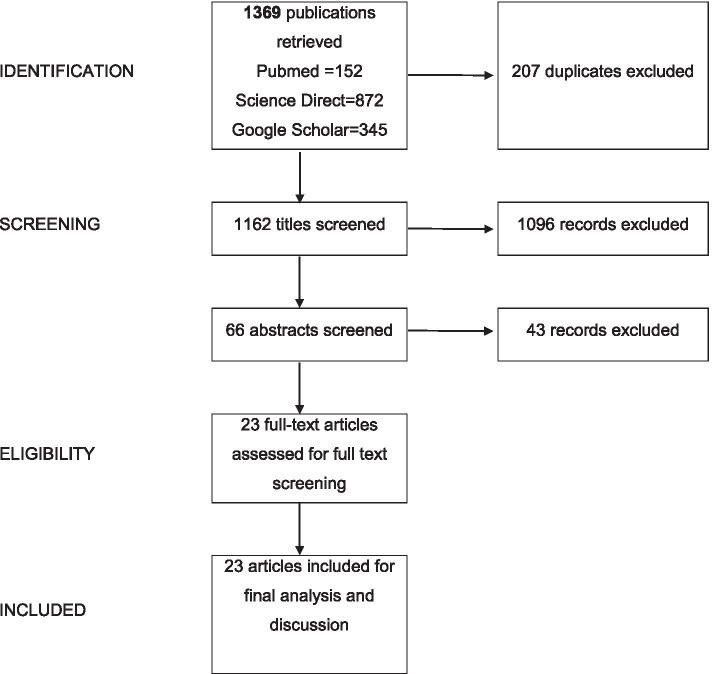
Study selection process

A total of 1162 articles remained after removing the duplicates. After screening of titles, 66 articles remained, and 23 articles were found eligible and were included for full-text assessment after abstract screening. No additional studies were added after further consultation and screening of reference lists (Fig. 1).

Two studies were rated as being of excellent quality (100% MMAT score), and the rest of the studies (*n* = 21) were rated as being of high quality (80% MMAT score) (Additional file 2). As indicated in Table 1, the NSSE reported after RP were collectively reported 27 times, whereas NSSE’s after RT were reported only 12 times.

**Table 1:** Studies reporting of specific NSSE after PCa treatment

NSSE after early PCa treatment after surgical and non-surgical intervention					
	27 studies	Surgical interventions (RP)	Non-surgical interventions (RT)	12 studies	
Reference	Number of studies	NSSE		Number of studies	Reference
[5, 20–24]	*6*	*Orgasmic dysfunction*		*1*	[4]
[5]	*1*	*Altered perception of orgasm*		*1*	[4]
[5, 24–26]	*4*	*Orgasm-associated pain*		*1*	[4]
[5, 27–30]	*5*	*Climacturia*		*2*	[4, 27]
	*0*	*Anejaculation*		*3*	[4, 31, 32]
[5]	*1*	*Penile sensory changes*		*1*	[4]
[5, 33–39]	*8*	*Penile length shortening*		*2*	[4, 36]
[5, 40]	*2*	*Penile deformity/Peyronie’s disease*		*1*	[4]

Frey et al. published two studies in 2014 and 2017 that reported all 8 NSSEs of interest in our review. The 2017 study reported on NSSE following RT interventions, and the 2014 study reported on the prevalence of NSSE after RP interventions [4, 5].

**Fig. 2:**
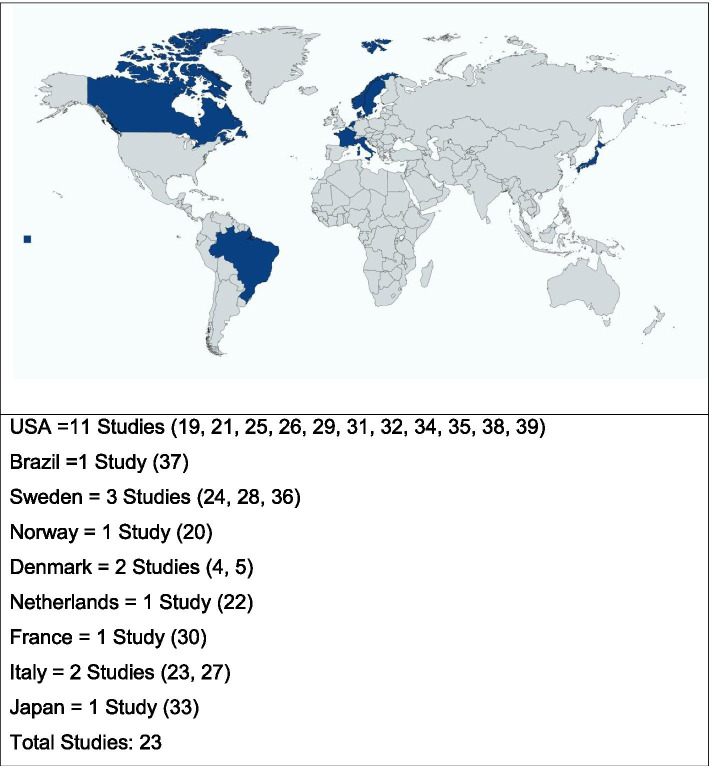
Distribution of study origin

All the studies included for review (*n* = 23) had cross-sectional study designs and specifically examined NSSEs after PCa treatment. A summary is provided in Table 2*.* The included studies represented data from 9 countries, with 11 of the studies having been conducted in the USA. Eleven of the remaining studies were conducted in European countries; one study was conducted in South America (Brazil), and one was in Asia (Japan). No African or Australasian studies matched the inclusion criteria (see Fig. 2.)

**Table 2:** Prevalence of NSSE

NSSE reported	First author/year/reference	Participant numbers/age	Time frame after intervention	Reported prevalence in the study population
Multiple	***Frey, 2017*** [4]	109 men (median age 71)	Three months to 5 years	24% reported anorgasmia 11% reported anejaculation 44% reported a decrease in orgasm intensity 4% reported urinary incontinence during sexual activity 40% reported an increased time needed to achieve orgasm 15% reported pain during orgasm 27% reported sensory changes in their penis 42% reported penile length shortening 12% reported an abnormal curve in the penis
Multiple	***Frey, 2014*** [5]	316 men (median age 64)	3–36 months	5% of the sexually active participants had reported anorgasmia 60% of the sexually active participants had reported a decrease in orgasm intensity 57% reported delayed orgasms 10% of sexually active participants had painful orgasms 38% reported urinary incontinence during sexual activity 25% reported sensory changes in their penis 47% reported a self-reported penile length loss of more than 1 cm 10% reported an abnormal curve in the penis
Orgasmic pain	***Mogorovich, 2013*** [25]	1288 men (median age 63)	Six months to 5 years	11% of participants reported a painful orgasm in the previous 6 months
Orgasmic pain	***Matsushita, 2012*** [26]	702 men (mean age 64)	6–24 months	12% of participants reported dysorgasmia
Orgasmic dysfunction	***Du, 2017*** [20]	415 men (median age 60)	36 months	60.2% of participants had a worse orgasmic function
Orgasmic dysfunction	***Ostby-Deglum, 2016*** [21]	609 men (median age 63)	Three years	78% of participants had poor ability to reach orgasm
Orgasmic dysfunction	***Tewari, 2012*** [22]	408 men (median age 60)	36 months	11.6% of participants under age 60 unable to achieve orgasm/17.4% over 60
Orgasmic dysfunction	***Dubbelman, 2010*** [23]	458 men (median age 64)	Up to 2 years	33.2% had orgasmic dysfunction afterwards with an age-related decline
Orgasmic dysfunction + pain	***Salonia, 2010*** [24]	334 men (median age 62)	Over 48 months	37% of participants reported complete inability to achieve orgasm, 14% of participants reported pain during orgasm
OAI/climacturia	***O'Neil, 2014*** [27]	412 men (mean age 62)	10–20.3 months	Climacturia was reported in 22.6% of the study group
OAI/climacturia	***Manassero, 2012*** [28]	*Seven men (mean age 64))*	One year	28.6% Climacturia reported as baseline investigations for a N/A study
OAI/climacturia	***Nilsson, 2011*** [29]	1261 men (median age 63)	Two years	21% of the participants had experienced orgasm-associated incontinence
Incontinence during sexual activity	***Mitchell, 2011*** [30]	1421 men (median age 58,4)	3–24 months	44% and 36.1% at 3 months and 24 months
Ejaculation function	***Sullivan, 2013*** [32]	364 men (median age 64)	Six years	72% lost the ability to ejaculate in an anterograde fashion
Ejaculatory function	***Huyghe, 2009*** [31]	198 men (median age 65)	36 months	18.7% had impaired ejaculatory function
Penile length shortening	***Kwon, 2018*** [33]	507 men (median age 59,3)	Seven days to 12 months	60.2% of the participants regained their pre-op penile length at 12 months
Penile length shortening	***Kadono, 2017*** [34]	102 men (median age 64,4)	Seven days to 24 months	MRI results concluded that the distal end of the membranous urethra moved proximally (mean proximal displacement of 3.9 mm) at 10 days after RP and then returned to the preoperative position at 12 months
Penile length shortening	***Berookhim, 2014*** [35]	118 Men (median age 58)	Baseline, 2 months, 6 months	2.4 mm difference (shortening) in stretched flaccid penis length compared to baseline, at 6 months, there was no difference compared to baseline
Penile length shortening	***Parekh, 2013*** [36]	948 (¾ of the participants = 60–80 years old)	Unavailable	3.73% of surgical cases had reduced penile length shortening, 0% RT cases
Penile length shortening	***Carlson, 2012*** [37]	1288 men (median age 64.8)	24.2 months	55% of participants had self-perceived penile length shortening.
Penile length shortening	***Vasconcelos, 2012*** [38]	105 men (median age 65)	3–60 months	1 cm mean penile length loss at 3 to 24 months, baseline penile length re-established at 48 months
Penile length shortening	***Engel, 2011*** [39]	127 men (median age 56.5)	1–11 months after	11.77 cm to 11.13 cm at 1 month after the surgery Mean stretched penile length was not significantly different from baseline at 9, 10 and 11 months
Penile length deformity/Peyronie’s disease	***Tal, 2010*** [40]	1011 men (median age 60.2)	Up to 3 years	Peyronie’s disease incidence, 15.9% in RP population, developed on average at 13.9 months, mean curvature magnitude was 31°

### Orgasmic dysfunction/anorgasmia (7 studies)

Six RP studies met the inclusion criteria [5, 20–24], whilst only one RT study reported on the prevalence of anorgasmia [4]. A low- to high prevalence range (5–78%) was reported between studies for orgasmic dysfunction. Two thirds of men reported poor ability to orgasm at 3 years [20, 21], and one third of men reported no orgasm at 2–5 years after an RP [23, 24]. Orgasmic function improved postoperatively with time [24], also deteriorated with age [20, 22–24]. Nerve-sparing RP procedures predicted better post-operative orgasmic function [20, 22]. Increased time needed to reach orgasm was experienced by almost half the men, 5 years after RT [4].

### Altered perception of orgasm (2 studies)

One RP study [5] and one RT study [4] reported decreased orgasm intensity. Similar results were found in the RP study and the RT study. The RP study [5] showed that 60% of participants and almost 50% of the RT participants reported decreased orgasm intensity [4].

### Orgasm-associated pain/dysorgasmia (5 studies)

Four studies included in this review reported on decreased orgasmic function after RP [5, 24–26] and one after RT [4]. Similar results were found between the RP studies, in that between 10 and 12% of RP participants reported orgasmic pain in RP [5, 25, 26]. The RT study reported a 15% prevalence of orgasmic pain in their study population [4].

### Orgasm-associated incontinence/climacturia (6 studies)

Four RP studies met the inclusion criteria [5, 27–30], and one RT study was included for climacturia [4]. One study reported on both RP and RT participants [27]. The prevalence was reported between 21% [29] to 38% [5] of participants across the five RP studies after 12–24 months (5, 27–30). The collaborative study recorded orgasm-associated incontinence/climacturia in 22.6% of the total study group (RP and RT participants), but the RT participants only represented 5.2% of the total participants [27]. The RT study reported a 4% prevalence of symptoms, but the symptoms were defined as urinary incontinence during sexual activity [4].

### Anejaculation (3 studies)

No RP studies in the current review reported this issue, and three RT studies were included [4, 31, 32]. Anejaculation worsened with time after RT in one study and peaked at 5 years after treatment, with 89% of the study group being affected [32]. An older study reported a conflicting rate of anejaculation, with 81.3% of their participants conserving their ejaculatory function [31]. This study reported that 75% of the participants had a reduction in ejaculate volume and that 19% of the men experienced dry ejaculation [31]. The final study reported an anejaculation prevalence of 11% in their study population [4]

### Penile sensory changes (2 studies)

Only one RP study [5] and one RT [4] study were included, with similar results being reported across the two studies. Penile sensory changes were reported in 25% of the RP study participants [5] and 27% of the RT study participants [4].

### Penile length shortening (10 studies)

Eight RP studies met the inclusion criteria for review [5, 33–39], and two RT studies [4, 36] studies were included for review. Only one study reported both on RT and RP and concluded that no RT participants had penile length shortening [36]. Penile length shortening was reportedly worse at 7–10 days postoperatively [33, 34] but started recovering at 3–6 months [39]. However, self-perceived penile length shortening was still experienced by 55% of men two years after RP [37]. Men who eventually did not fully regain their penile length had experienced up to a 24% loss in length at 7 days postoperatively [33]. The second RT study reported that 42% of participants reported more than 1 cm subjective penile length shortening [4].

### Penile deformity/Peyronie’s disease (3 studies)

Two RP studies [5, 40] and one RT study [4] were included for review. Ten per cent of participants in a 2014 study were found to have an abnormal curvature of their penis [5]. Two studies on RP participants found that 10–15.9% of participants reported the presence of penile curvature or penile deformity [5, 40]. The average reported curvature angle was 31° [40]. A similar result was reported in the only RT study, where 12% of the participants reported an altered curve of the penis [4].

#### Questionnaire use in NSSE studies

The included studies used a variety of questionnaires that included validated and non-validated questionnaires. Some studies included a mixed-method design and added either an interview or a physical examination component to the questionnaire. Table 3 outlines how questionnaires were used in the included studies.

**Table 3:** Questionnaire used after early PCa treatment

NSSE reported	First author, year, reference	Questionnaire used to report NSSE
Multiple	***Frey, 2017*** [4]	Study-specific questionnaire based on various other questionnaires and tools, including the Erection Hardness Scale and International Consultation of Incontinence-Short Form
Multiple	***Frey, 2014*** [5]	Study-specific questionnaire based on various other questionnaires and tools including the International Index of Erectile Function, International Consultation of Incontinence-Short Form and Erection Hardiness Scale
Orgasmic pain	***Mogorovich, 2013*** [25]	Study-specific questionnaire consisting of 145 questions—5 pertaining to orgasmic characteristics
Orgasmic pain	***Matsushita, 2012*** [26]	Dysorgasmia Frequency Scale and Visual Analogue Scale
Orgasmic dysfunction	***Du et, 2017*** [20]	Expanded Prostate Index Composite, American Urological Association Symptom Index and Sexual Health Inventory for Men. Participants were asked to rate their post-operative orgasmic function
Orgasmic dysfunction	***Ostby-Deglum, 2016*** [21]	Expanded Prostate Index Composite 26—one single question asked
Orgasmic dysfunction	***Tewari, 2012*** [22]	Health-Related Quality of Life questionnaire, Expanded Prostate Index Composite and International Index of Erectile Function. Participants were asked to rate their post-operative orgasmic function
Orgasmic dysfunction	***Dubbelman, 2010*** [23]	N/A
Orgasmic dysfunction	***Salonia, 2010*** [24]	International Index of Erectile Function and International Consultation of Incontinence -Short Form. Structured Interviews
Orgasm-associated incontinence/climacturia	***O'Neil, 2014*** [27]	A non-validated questionnaire was used
Orgasm-associated incontinence/climacturia	***Manassero, 2012*** [28]	International Index of Erectile Function (5 Item) and International Prostate Symptom Score. Telephonic interview about orgasm-associated incontinence/climacturia
Orgasm-associated urinary incontinence	***Nilsson, 2011*** [29]	The author designed a study-specific questionnaire based on the Scandinavian prostate cancer group 4 questionnaire.
Incontinence during sexual activity	***Mitchell, 2011*** [30]	The University of California and Los Angeles Prostate Cancer Index.
Ejaculation function	***Sullivan, 2013*** [32]	International Index of Erectile Dysfunction
Ejaculatory function	***Huyghe, 2009*** [31]	The author designed a study-specific questionnaire based on an adapted Male Sexual Health questionnaire
Penile length shortening	***Kwon, 2018*** [33]	Sexual Health Inventory for Men and Physical measurement
Penile length shortening	***Kadono, 2017*** [34]	International Index of Erectile Function and Erection Hardness Score. The physical exam using a ruler to measure stretched flaccid penile length
Penile length shortening	***Berookhim, 2014*** [35]	International Index of Erectile Function questionnaire. Physical exam to measure stretched flaccid penile length
Penile length shortening	***Parekh, 2013*** [36]	A non-validated questionnaire was used
Penile length shortening	***Carlsson, 2012*** [37]	The author designed a study-specific questionnaire based on previous work of the study group
Penile length Shortening	***Vasconcelos, 2012*** [38]	International Index of Erectile Function. Physical Assessment
Penile length shortening	***Engel, 2011*** [39]	International Index of Erectile Function. The physical exam using a semi-rigid ruler to measure stretched flaccid penile length
Peyronie’s disease	***Tal, 2010*** [40]	Descriptive statistics. Physical examination with a goniometer

## Discussion

The NSSE after PCa treatment has gained some attention over the last few years. However, more attention is given to individual NSSE rather than the collective group, and more studies focus on the NSSE related to RP than RT. Comparisons across studies were limited as different methodologies, assessment time frames, varying treatment approaches, and the use of non-validated questionnaires varied and impacted the criteria for comparisons.

### Prevalence of NSSE

Orgasmic dysfunction had a low to high prevalence. However, it was almost exclusively reported in RP studies (5–78%), except for one RT study reporting a 24% prevalence amongst their participants [4]. Possible reasons for the considerable variation in the results across studies may be due to the variable lengths of time reported after the intervention, participant age, nerve sparing status and various methods/questionnaires to determine orgasmic dysfunction. This observation concurs with a 2014 systematic review where 80% of RP patients were reported to have some degree of orgasmic dysfunction after RP with similar variables influencing the prevalence [7].

Altered perception of orgasm showed a similar moderate prevalence (50–60%) between RP and RT studies [4, 5]. Orgasmic pain similarly showed a low prevalence (10–15%) between RP and RT studies [4, 5, 25, 26, 41]. One study further described that the orgasmic pain felt mainly (70% of the time) was felt in the penis [26]. At the same time, another made the association between bilateral seminal vesicle sparing procedures as a possible cause of orgasmic pain [25]. This notion was concurred in the systematic review by Frey et al., who reported that sparing the tips of the seminal vesicles doubles the risk of orgasmic pain [7].

Penile length changes showed a low to moderate prevalence (0**–**55%) after RP and RT [4, 5, 33–39]. Nerve-sparing procedures reportedly reduced the risk of self-perceived penile length shortening [37], whilst younger age and better preoperative erectile function were associated with complete penile length recovery [33]. Penile length shortening was also associated with treatment regret [36]. Furthermore, the self-perceived penile length shortening was found to be much more than actual penile length shortening measured using a ruler [37]. The study by Parekh et al. is of particular interest as an outlier study, as they only reported a 3.73% RP and a 0% RT prevalence of penile length shortening [36]. This study relied on self-reported patient outcomes, but participants were not instructed on the required measuring procedures (stretched or relaxed flaccid penile length or erect penile length). Furthermore, the majority of the participants (75.4%) in Park et al.’s study were aged between 60 and 80 years old. The lack of available baseline data compromised the ability to determine penile length loss objectively. Frey et al. reported a 15**–**68% prevalence of penile length shortening in their study [7], placing the results of a 42% (RT study) [4] and 47% (RP study) [5] more within the expected range.

Penile curvature changes were also similar between RP and RT studies, showing a low prevalence (10–15.9%) [4, 5, 40], and the average reported abnormal penile curvature angle was 31° [40]. Penile sensory changes showed an almost similar moderate prevalence between RP (25%) and RT (27%) participants [4, 5].

Anejaculation was found to have a low to high prevalence (11–72%) after RT [4, 31, 32]. According to this review, anejaculation is a consequence of RT [31, 32], and it is at its worst 5 years after treatment [32]. Conserved ejaculatory function is often associated with a reduction in ejaculate volume. Higher RT dose, older age and smaller prostates at the time of treatment increased the likelihood of failure to ejaculate [32]. Anejaculation is, however, also a given consequence of RP, as the ejaculatory apparatus (prostate, seminal vesicles and ejaculatory ducts) are removed [7, 42]. However, the authors could not source any studies within our search parameters that met the study inclusion criteria.

Climacturia has a reported moderate prevalence (21–38%) after RP [5, 27–30] and a low prevalence (4–5.2%) after RT [4, 27]. A comparative study concluded that the orgasm-associated incontinence rates after RP were six times more than that of RT (28.3% vs 5.2%) [27]. Climacturia is associated with major sexual inconvenience and bother [29].

### Questionnaire used in assessing NSSE

None of the retrieved studies reported on a validated, standardised questionnaire to investigate the NSSE after early PCa treatment. Most studies incorporated either some aspects of other questionnaires or designed their own. Two studies used a non-validated questionnaire that was able to identify the majority of the collective group of NSSE [4, 5]. This questionnaire enquired about orgasmic dysfunction, orgasm-associated pain, climacturia, penile sensory changes, penile length shortening and penile deformity. These two studies looked mainly and the prevalence and predicting factors of the NSSE.

Interestingly, a limited number of studies reportedly described the use of the Expanded Prostate Cancer Index questionnaire [11] to gather patient data relating to orgasmic dysfunction [20–22]. However, the Expanded Prostate Cancer Index questionnaire was inadequate to report on the NSSE, and additional questions that inquired into orgasmic function were added [20, 22]. The Expanded Prostate Cancer Index-26 questionnaire was similarly inadequate to detect NSSE. It merely asked respondents to “rate their ability to reach orgasm” without exploring any symptoms relating to the other NSSE [21].

A 2011 study used the Expanded Prostate Cancer Index questionnaire similarly at regular intervals after surgery to investigate orgasmic outcomes [22]. In addition, patients were asked to evaluate their orgasm and state whether they experienced any pain during orgasms. One study also incorporated the Dysorgasmia Frequency Scale [26]. The International Index Erectile Function was used in many studies [5, 22, 24, 28, 32, 34, 35, 38, 39] but served no purpose in detecting any of the NSSE. The Erection Hardness Scale [43] was used in a few studies [5, 34] and had no role in detecting the NSSE. The Sexual Health Inventory for Men questionnaire (a modified 5-item version of the International Index Erectile Function) was used in two studies [20, 33], and another study [31] based their informal questionnaire on the Male Sexual Health Questionnaire [44].

Orgasm-associated incontinence/climacturia was further assessed by a non-validated author designed questionnaire [27] and a study-specific questionnaire based on the Scandinavian Prostate Cancer Group 4 questionnaire [29] in two separate studies. A telephonic interview was added to a non-NSSE questionnaire to probe the presence of climacturia in a 2012 study [28].

Anejaculation was assessed in a study that used the International Index of Erectile Function questionnaire [32]. A sexual medicine physician initially interviewed the participants. They were then questioned about their ejaculatory function (presence/absence, intensity and ease of achievement) and orgasm (presence/absence, intensity and ease of achievement). Only those who were sexually active were asked to complete the questionnaire. Questions 9 and 10 respectively asked: “When you had sexual stimulation or intercourse, how often did you ejaculate?” and “When you had sexual stimulation or intercourse, how often did you have the feeling of orgasm or climax?” [32]. A 2009 study used a modified version (5 items, not 7) of the Male Sexual Health questionnaire that specifically addressed: (i) frequency, (ii) volume, (iii) dryness, (iv) pleasure and (v) pain during ejaculation [31].

Penile length shortening was assessed in a 2012 study using an author designed questionnaire containing questions relating to self-perceived penile length shortening [37]. Penile length shortening and penile deformity/Peyronie’s disease were not assessed by any other questionnaires apart from the collective NSSE questionnaire mentioned [4, 5], but rather through physical examinations. Three studies used a semi-rigid ruler for a physical penile length examination [34, 35, 39]. Vasconcelos et al. used an anthropometric ruler as a physical measurement to assess shortening [38]. Parekh et al. reported in their study that physicians completed a questionnaire based on their patients, and one question includes under “the complaints section” referred to reduced penile length [36].

Penile deformity was assessed in one additional study by Tal et al., where they assessed a penile curvature with a goniometer if the patient reported an abnormal curvature [40].

### Strengths and limitations of the study

The methodology used and the search period used allowed for the systematic and extensive literature search, which sought to map only the most recent developments on the prevalence of NSSE and the use of questionnaires to identify NSSE. Additionally, the scoping review results were presented following the PRISMA recommendations, which ensured complete and transparent reporting. The MMAT tool version 2011 was used to assess the methodological quality of the included studies.

Limitations of this study included the fact that the studies included variables that were not consistent between studies. The reader should be cautioned when interpreting the results of the prevalence indicators for different NSSEs.

Furthermore, only original research was included, and other sources of information could have further clarified some discrepancies in the results.

## Conclusion

This study found a low to a high prevalence of NSSE reported in men after RP and RT. Penile deformity, orgasmic dysfunction, and penile length shortening were low to moderately prevalent, similar to RP and RT. Anejaculation prevalence was low to high after RT. Climacturia was shown to have a low prevalence after RT and a moderate prevalence after RP (six times more than RT). A common theme through most of the studies was that the participants expressed not being adequately informed about the possible sexual side effects before commencing their PCa treatment. Questionnaires effectively assess sexual dysfunction, and many modified informal non-specific questionnaires are used to detect conditions related to sexual dysfunction. There is currently no valid and reliable questionnaire to detect the collective NSSE after PCa treatment. There is a need to develop a validated and reliable NSSE questionnaire for use after PCa treatment for quick and effective diagnosis.

## Supplementary Information

**Additional file 1.** Collected data organised into subgroups.

**Additional file 2.** The specific criteria to determine the appropriateness of each included study.

**Additional file 3.** Preferred Reporting Items for Systematic reviews and Meta-Analyses extension for Scoping Reviews (PRISMA-ScR) Checklist.

## Data Availability

Not applicable

## Abbreviations

PCa: Prostate cancer
NSSE: Neglected sexual side effects
PCC: Population concept context
MeSH: Medical Subject Heading
MMAT: Mixed Method Appraisal Tool
PRISMA-ScR: Preferred Reporting Items for Systematic Reviews and Meta-Analysis Extension for Scoping Reviews
RP: Radical prostatectomy
RT: Radiation therapy
