# Supplementary material for: Mapping the prevalence and use of questionnaires to detect the neglected sexual side effects after prostate cancer treatment: a scoping review
Source: Syst Rev. 2022 Jan 3;11:2. doi: 10.1186/s13643-021-01865-5 (PMC8722282; doi:10.1186/s13643-021-01865-5)
Supplement: Supplementary file 1 — Additional file 1. Collected data organised into subgroups. [file 13643_2021_1865_MOESM1_ESM.docx]

Additional file 1

| APPENDIX 1 | | | | | | | | | |
| --- | --- | --- | --- | --- | --- | --- | --- | --- | --- |
| First author/year/Study Title/Reference | Study Design | Geographical Setting | Number of Participants, Age at treatment intervention and Intervention reported | NSSE Reported | Period after treatment  investigated | Aims and Objectives | NSSE Prevalence Reported | Questionnaire Used to Report NSSE | Quality of Study (MMAT) |
| ***Frey, 2017***  Prevalence and Predicting Factors for Commonly Neglected Sexual Side Effects to External-Beam Radiation Therapy for Prostate Cancer (1) | Quantitative:  Questionnaire based investigation | Denmark (single Center) | 109 men (median age 71) treated with  RT-External Beam | Multiple | 3 Months to 5 years | To investigate orgasmic dysfunction, urinary incontinence during sexual activity, changes in penile morphology and penile sensory changes. | ***Of the study participants***  24% reported anorgasmia  11% reported anejaculation  44% reported a decrease in orgasm intensity  4% reported urinary incontinence during sexual activity  40% reported an increased time needed to achieve orgasm  15% reported pain during orgasm  27% sensory changes in their penis  42% reported penile length shortening  12% reported an abnormal curve in the penis | Study specific questionnaire based on various other questionnaires and tools, including the EHS, ICIQ-SF | Good quality (80%) where most of the criteria is met |
| ***Frey, 2014***  Prevalence and Predicting Factors for Commonly Neglected Sexual Side Effects to Radical Prostatectomies:Results from a Cross Sectional Questionairre based study (2) | Quantitative:  Questionnaire based investigation | Denmark | 316 men (median age 64) reated with RP | Multiple | 3-36 Months | Prevelence and predicting Factors for Coomonly Neglected Sexual side Effects to radical prostatectomy | ***Of the study participants***  5% of the sexually active participants had reported anorgasmia  60% of the sexually active participants had reported a decrease in orgasm intensity  57% reported delayed orgasms  10% of sexually active participants had painful orgasms  38% reported urinary incontinence during sexual activity  25% reported sensory changes in their penis  47% reported a self-reported penile length loss of more than 1 cm  10% reported an abnormal curve in the penis | Study specific questionnaire based on various other questionnaires and tools including the IIEF, ICIQ-SF and the EHS. | Good quality (80%) where most of the criteria is met |
| ***Mogorovich, 2013***  Radical prostatectomy, sparing of the seminal vesicles, and painful orgasm (3) | Quantitative  Questionnaire based investigation | Sweden | 1288 men (median age 63) treated with RP:  RRP (33%) and RALP (67%) | Orgasmic Pain | 6 months to 5 years | To investigate the prevalence of painful orgasm and to identify potential risk factors | 11% of participants reported a painful orgasm in the previous 6 months  21% of bilateral seminal vesicle sparing participants reported PO  12 % of unilateral seminal vesicle participants reported PO  ***Other Findings***  Bilateral seminal vesicle sparring and age below 60 significantly related to the presence of painful orgasm. | Study specific questionnaire consisting of 145 questions-5 pertaining to orgasmic characteristics | Good quality (80%) where most of the criteria is met |
| ***Matsushita, 2012***  The Evolution of Orgasmic Pain (Dysorgasmia) Following Radical Prostatectomy (4) | Quantitative  Questionnaire based investigation | USA | 702 men (mean age 64) treated with RP | Orgasmic pain | 6-24 months | To Assess the evolution of dysogasmia in RP Patients | 12% of participants reported dysorgasmia  72%, 26% and 7% of participants complained of pain during orgasm at 12, 18 and 24 Months  An intensity of pain of 6/10 or more was experienced by 26% of participants at 6 months, and 165 of participants at 24 months.  ***Other Findings***  70% reported PO in the Penis, 22% in testes, 8% other areas. | Dysorgasmia Frequency Scale  VAS | Good quality (80%) where most of the criteria is met |
| ***Du, 2017***  Orgasmic Function after Radical Prostatectomy (5) | Quantitative  Questionnaire based investigation | USA (Single center) | 415 men (median age 60) treated with RP  (median age unavailable) | Orgasmic Dysfunction | 36 months | To examine OF overtime after RP | 60,2% of participants had OF worsening after the RP, whereas 30,5% remained stable, and 9,4% improved.  High pre-operative OF predicted high post-operative OF (15 x that of low OF pre-operative men)  Younger age (below 50) and nerve sparing procedures predicted higher post-operative OF | EPIC  AUASI  SHIM  ***Plus***  **Asked to rate their post-operative orgasmic function** | Good quality (80%) where most of the criteria is met |
| ***Ostby-Deglum, 2016***  Ability to Reach Orgasm in Patients With Prostate Cancer Treated With Robot-assisted Laparoscopic Prostatectomy (6) | Quantitative  Questionnaire based investigation | Norway | 609 men (median age 63) treated with RP:  RALP | Orgasmic Dysfunction | 3 years | To study the ability to reach orgasm after RALP | 78% of participants had poor ability to reach orgasm  23% of participants reported good ability to reach orgasm  Older age, poor physical quality of life and erectile dysfunction associated with poor orgasmic ability | EPIC 26- one single question | Good quality (80%) where most of the criteria is met |
| ***Tewari, 2012***  Nerve sparing can preserve orgasmic function in most men after robotic-assisted laparoscopic radical prostatectomy (7) | Quantitative  Questionnaire based investigation | USA | 408 men (median age 60) treated with RP:  RALP | Orgasmic Dysfunction | 36 Months | To investigate orgasmic outcomes in patients undergoing RALP and the effects of age and nerve sparring on these outcomes | 88,4% of participants under age 60 able to achieve orgasm/82,6% over 60  90,7% BNS participants able to achieve orgasm/82,1 UNS  3,2% of BNS had decreased sensation of orgasm | HRQOL  EPIC  IIEF  ***Plus***  **Asked to rate their post-operative orgasmic function** | Good quality (80%) where most of the criteria is met |
| ***Dubbelman, 2010***  Orgasmic dysfunction after open radical prostatectomy: clinical correlates and prognostic factors (8) | Quantitative:  Analysis of clinical notes | Netherlands | 458 men (median age 64) treated with RP: RRP (over a 30-year period. | Orgasmic Dysfunction | Up to 2 years | To analyse sexual function and orgasmic function in men before and after RP | Of the studied men who had orgasmic function before the surgery, 66,8% had orgasmic function afterwards with an age-related decline | N/A | Good quality (80%) where most of the criteria is met |
| ***Salonia, 2010***  Postoperative orgasmic function increases over time in patients undergoing nerve-sparing radical prostatectomy (9) | Mixed method design | Italy | 334 men (median age 62) treated with RP: BNSRRP | Orgasmic Dysfunction | Over 48 months | To assess orgasmic functioning over time in men after bilateral nerve sparing-sparring radial retro pubic prostatectomy for PCa | 37% of participants reported complete inability to achieve orgasm  14% of participants reported pain during orgasm  Orgasmic function showed an increase over time postoperatively  Orgasmic function deteriorated with age postoperatively | IIEF  ICIQ-SF  ***Plus***  ***Structured Interviews*** | Good quality (80%) where most of the criteria is met |
| ***O’Neil, 2014***  Climacturia after definitive treatment of prostate cancer (10) | Quantitative  Questionnaire based investigation | USA | 412 men (mean age 62,2 (RP), 65 (RT) and Combined approaches 63,2)  RP (67,7% of which 39,1 % open procedure vs robotic, and 88,45 underwent a nerve sparing procedure)  RT (26,7%, of which 45,5% Brachytherapy, 34,5 % external beam radiation and 20% combined)  RP+RT (5,6%) | OAI/Climacturia | RP: 20,3 Months  RT: 23,9 Months  RP+RT: 10,2 Months | To examine the prevalence, causes and impact on orgasm function of climacturia after definitive treatment of PCa with surgery | Climacturia reported in 22.6% of study group  Climacturia was more prevalent after surgery (28,3%) compared to radiation (5,2%).  Relationship between Stress Urinary Incontinence and Climacturia  Climacturia is not necessarily associated with negative sexual satisfaction | Non-validated questionnaire was used | Good quality (80%) where most of the criteria is met |
| ***Manassero, 2012***  Orgasm-associated incontinence (climacturia) after bladder neck-sparing radical prostatectomy: clinical and video-urodynamic evaluation (11) | Quantitative | Italy | Phase 1: 84 men  ***Phase 2: 7 men (mean age 64,1) underwent video analysis-N/A*** | OAI/Climacturia | 1 year | To Investigate the incidence and Video-urodynamics of Climacturia in continent and potent patients after BNS RP | 28,6 % Climacturia reported as baseline investigations for a N/A study | IIEF (5 Item)  International Prostate Symptom score  ***Plus Telephonic interview about climacturia*** | Good quality (80%) where most of the criteria is met |
| ***Nilsson, 2011***  Orgasm-associated urinary incontinence and sexual life after radical prostatectomy (12) | Quantitative | Sweden | 1261 men (median age 63) treated with RP | Orgasm Associated urinary incontinence | Mean follow up of 2 years after RP | To determine the prevalence of OAI after RP and its effect on sexual satisfaction | 45% of the study participants were not sexually active  21% of the participants had experienced OAI  Of the sexually active men:  4% had urinary leakage during every orgasm, 39% have had an incident of urinary leakage during orgasm, and 7% of participants experienced OAI more than half the time.  *Other findings*  orgasm associated urinary incontinence in the previous 6 months was associated with   1. decreased ability to satisfy sexual partner, 2. avoiding sexual activity due to fear of failing 3. inferior orgasmic satisfaction 4. lower frequency of sexual intercourse | Author designed study specific questionnaire based on the Scandinavian prostate cancer group 4 questionnaire. | Good quality (80%) where most of the criteria is met |
| ***Mitchell, 2011***  ost-prostatectomy incontinence during sexual activity: a single center prevalence study. (13) | Quantitative | USA | 1421 men (median age 58,4) treated with RP (82,7% BNS, 14,6 % UNS, 2,7% none) | Incontinence during sexual activity | 3, 6, 14 and 24 months | To determine the time dependent prevalence and severity of incontinence during sexual activity | 44% of participants reported Incontinence during sexual activity at 3 months  36,1% of participants reported Incontinence during sexual activity at 24 Months | UCLA PCI | Good quality (80%) where most of the criteria is met |
| ***Sullivan, 2013***  Ejaculation Profiles of Men Following Radiation Therapy for Prostate Cancer (14) | Quantitative: Structured interview and questionnaire | USA | 364 men (median age 64) treated with RT: (68,6% External Beam and 30,7% Brachytherapy) | Ejaculation function | 6 years | To define the ejaculation profiles of men after RT for PCa. | 72% of the study population lost the ability to ejaculate in an anterograde fashion  Anejaculation was experienced at 1,3 and 5 years after RT by 16%, 69% and 89% of the respondents  *Other Findings*  The likelihood of failure to ejaculate is increased in men with a high dose of RT, older age, and smaller prostates at the time of RT. | International Index of Erectile Dysfunction Questionnaire | Good quality (80%) where most of the criteria is met |
| ***Huyghe, 2009***  Ejaculatory Function After Permanent 125I Prostate Brachytherapy for Localized Prostate Cancer (15) | Quantitative:  Questionnaire based investigation  To conduct a detailed analysis of ejaculatory function after brachytherapy for PCa | France | 241 men before and 198 men after treatment (median age 65) treated with RT (brachytherapy) | Ejaculatory Function | 36 months | To conduct a detailed analysis of ejaculatory function after brachytherapy for PCa | - Most participants (81,3%) had conserved ejaculatory function at 36 months after prostate brachytherapy. - ¾ of the men with conserved ejaculatory function after the treatment experienced a reduction in ejaculate volume ( - 18,7 % of the sexually active respondents experienced dry ejaculation after treatment - 12,9% had painful ejaculations before, compared to 30,3% after - 10% of respondents experience no orgasms during sexual activity after treatment compared to only 1% before. | Author designed study specific Questionnaire based on an adapted Male Sexual Health Questionnaire | Good quality (80%) where most of the criteria is met |
| ***Kwon, 2018***  Longitudinal recovery patterns of penile length and the underexplored benefit of long-term phosphodiesterase-5 inhibitor use after radical prostatectomy (16) | Quantitative study | USA | 507 men (median age 59,3% CR group and 62 IR group) treated with RP | Penile length shortening | 7 days-12 months | To describe longitudinal patterns of Pl recovery and to evaluate factors predicting complete return to baseline PL | The CR group experienced an average 14,13% (1,9cm) reduction in PL at 7 days after the RD  The IR group experienced a 23,8 % (3,38cm) reduction in PL at 7 days after the RP  60,2 % of the participants regained their pre-op PL at 12 months  Younger age and pre-operative Erectile function are associated with complete PL recovery. | Physical measurement  SHIM | Good quality (80%) where most of the criteria is met |
| ***Kadono, 2017***  Changes in penile length after radical prostatectomy: investigation of the underlying anatomical mechanism (17) | Quantitative:  Questionnaire based | Japan | 102 men (median age 64,4) treated with RP | Penile length shortening | Before RP  7 days-24 months after RP | To measure changes in penile length over time before and after RP, and to investigate the underlying mechanisms for these changes. | - Stretched penile length was shortest at 10 days (mean shortening of 19,9 mm) after RP and gradually recovered thereafter - Stretched penile length at 12 months after RP was not significantly different to that of the preoperative measurement   MRI results concluded that the distal end of the membranous urethra moved proximally (mean proximal displacement of 3,9mm) at 10 days after RP, and then returned to the preoperative position at 12 months | International Index of Erectile Function Questionnaire and the Erection Hardness Score and a physical exam using a ruler to measure stretched flaccid penile length | Good quality (80%) where most of the criteria is met |
| ***Berookhim, (2014***  Prospective analysis of penile length changes after radical prostatectomy (18) | Qualitative | USA | 118 Men (median age 58) treated with RP | Penile length shortening | Baseline, 2months, 6 months | To assess the impact of RP on penile dimensions, and the assess the impact of Phosphodiesterase-5-inhibitor use on penile length changes | - At 2 months post operatively, there was a 2.4mm difference (shortening) in stretched flaccid penis length compared to baseline - At 6 months, there was no difference compared to baseline - PDE51 use preserved stretched flaccid penile length at 6 months | International Index of Erectile Function Questionnaire and a physical exam to measure stretched flaccid penile length | Good quality (80%) where most of the criteria is met |
| ***Parekh, 2013***  Reduced Penile Size and Treatment Regret in Men With Recurrent Prostate Cancer After Surgery, Radiotherapy Plus Androgen Deprivation, or Radiotherapy Alone. (19) | Quantitative | USA | 948 men treated for PCa  53,8% had RP  22.5 % had RT  23,7% had RT and ADT  ¾ of the participants were between 60-80 years old | Penile length shortening |  | To report the relative incidence of the perceived reduction in penile size across prostate cancer treatment modalities. | 3.73 % of Surgical Cases had reduced PL  2,67 % RT cases plus ADT had reduced PL  0% RT cases without ADT had reduced PL  Penile length shortening is associated increased treatment regret. | Non-validated questionnaire was used | Good quality (80%) where most of the criteria is met |
| ***Carlsson, 2012***  Self-perceived penile shortening after radical prostatectomy (20) | Quantitative:  Questionnaire based investigation | Sweden (Single center) | 1288 men (median age 64,8) treated with RP | Penile length shortening | 24,2 months | To evaluate self-perceived penile shortening in PCa after RP | 55% of RP Participants had Self Perceived PLS  With increasing nerve sparring operative procedure, the risk of self-perceived PLS decreased.  A subgroup of participants data reported that with an increase in nerve sparing operative procedures, there is a decrease in self perceived PLS with 58% of UNS participants reporting PLS compared to 33% BNS participants. | Author designed study specific questionnaire based on previous work of the study group | Good quality (80%) where most of the criteria is met |
| ***Vasconcelos, 2012***  The natural history of penile length after radical prostatectomy: a long-term prospective study (21) | Quantitative | Brazil | 105 men (median age 65) treated with RP | Penile Length Shortening | Baseline preoperatively  3-60 Months | To describe the penile length after RP in a long-term follow up | 1 cm mean PL loss at 3 to 24 months  Baseline PL reestablished at 48 months  Preserved sexual function will ensure baseline PL is reached sooner | IIEF  Physical Assessment | Good quality (80%) where most of the criteria is met |
| ***Engel, 2011***  Changes in Penile Length After Robot-Assisted Laparoscopic Radical Prostatectomy (22) | Quantitative:  Questionnaire based investigation and a physical assessment | USA | 127 men (median age 56,5) treated with RALP RP | Penile length shortening | Baseline, preoperatively, 1-11 months after | To describe changes in penile length  after robot assisted radical prostatectomy | - Stretched penile length reduced from a mean 11.77cm to 11.13cm at 1 month after the surgery - Recovery trends started at 3 months and 6 months - Mean stretched penile length was not significantly different from baseline at 9, 10 and 11 months | International Index of Erectile Function Questionnaire and a physical exam using a semi rigid ruler to measure stretched flaccid penile length | Excellent quality (100%) all criteria are met |
| ***Tal, 2010***  Peyronie's Disease Following Radical Prostatectomy: Incidence and Predictors (23) | Mixed method  Qualitative and Quantitative | USA | 1011 men (median age 60,2) treated with RP | Peyronies disease | Baseline, up to 3 years after RP | To define the incidence of PD in men who had RP and determine possible predictors of PD development after RP | PD Incidence 15,9 % in RP population  PD developed on average at 13,9 Months  Mean curvature magnitude was 31 Degrees  PD after a RP is more prevalent in young white men | Descriptive statistics  Physical Exam-goniometer | Good quality (80%) where most of the criteria is met |

1. Frey A, Pedersen C, Lindberg H, Bisbjerg R, Sonksen J, Fode M. Prevalence and Predicting Factors for Commonly Neglected Sexual Side Effects to External-Beam Radiation Therapy for Prostate Cancer. The journal of sexual medicine. 2017;14(4):558-65.

2. Frey AS, J.: Jakobsen, H.: Fode, M. Prevalence and Predicting Factors for Commonly Neglected Sexual Side Effects to Radical Prostatectomies: Results from a Cross-Sectional Questionnaire-Based Study. Journal of Sexual Medicine. 2014;11(9):2318-26.

3. Mogorovich A, Nilsson AE, Tyritzis SI, Carlsson S, Jonsson M, Haendler L, et al. Radical prostatectomy, sparing of the seminal vesicles, and painful orgasm. The journal of sexual medicine. 2013;10(5):1417-23.

4. Matsushita K, Tal R, Mulhall JP. The Evolution of Orgasmic Pain (Dysorgasmia) Following Radical Prostatectomy. The journal of sexual medicine. 2012;9(5):1454-8.

5. Du K, Zhang C, Presson AP, Tward JD, Brant WO, Dechet CB. Orgasmic Function after Radical Prostatectomy. J Urol. 2017;198(2):407-13.

6. Ostby-Deglum M, Axcrona K, Brennhovd B, Dahl AA. Ability to Reach Orgasm in Patients With Prostate Cancer Treated With Robot-assisted Laparoscopic Prostatectomy. Urology. 2016;92:38-43.

7. Tewari A, Grover S, Sooriakumaran P, Srivastava A, Rao S, Gupta A, et al. Nerve sparing can preserve orgasmic function in most men after robotic-assisted laparoscopic radical prostatectomy. BJU International. 2012;109(4):596-602.

8. Dubbelman Y, Wildhagen M, Schroder F, Bangma C, Dohle G. Orgasmic dysfunction after open radical prostatectomy: clinical correlates and prognostic factors. The journal of sexual medicine. 2010;7(3):1216-23.

9. Salonia A, Gallina A, Briganti A, Colombo R, Bertini R, Da Pozzo LF, et al. Postoperative orgasmic function increases over time in patients undergoing nerve-sparing radical prostatectomy. The journal of sexual medicine. 2010;7(1 Pt 1):149-55.

10. O'Neil BB, Presson A, Gannon J, Stephenson RA, Lowrance W, Dechet CB, et al. Climacturia after definitive treatment of prostate cancer. J Urol. 2014;191(1):159-63.

11. Manassero F, Di Paola G, Paperini D, Mogorovich A, Pistolesi D, Valent F, et al. Orgasm-associated incontinence (climacturia) after bladder neck-sparing radical prostatectomy: clinical and video-urodynamic evaluation. The journal of sexual medicine. 2012;9(8):2150-6.

12. Nilsson AE, Carlsson S, Johansson E, Jonsson MN, Adding C, Nyberg T, et al. Orgasm-associated urinary incontinence and sexual life after radical prostatectomy. The journal of sexual medicine. 2011;8(9):2632-9.

13. Mitchell SA, Jain RK, Laze J, Lepor H. Post-prostatectomy incontinence during sexual activity: a single center prevalence study. J Urol. 2011;186(3):982-5.

14. Sullivan JF, Stember DS, Deveci S, Akin‐Olugbade Y, Mulhall JP. Ejaculation Profiles of Men Following Radiation Therapy for Prostate Cancer. The journal of sexual medicine. 2013;10(5):1410-6.

15. Huyghe E, Delannes M, Wagner F, Delaunay B, Nohra J, Thoulouzan M, et al. Ejaculatory Function After Permanent 125I Prostate Brachytherapy for Localized Prostate Cancer. International Journal of Radiation Oncology*Biology*Physics. 2009;74(1):126-32.

16. Kwon YS, Farber N, Yu JW, Rhee K, Han C, Ney P, et al. Longitudinal recovery patterns of penile length and the underexplored benefit of long-term phosphodiesterase-5 inhibitor use after radical prostatectomy. BMC Urology. 2018;18(1).

17. Kadono Y, Machioka K, Nakashima K, Iijima M, Shigehara K, Nohara T, et al. Changes in penile length after radical prostatectomy: investigation of the underlying anatomical mechanism. BJU International. 2017;120(2):293-9.

18. Berookhim BM, Nelson CJ, Kunzel B, Mulhall JP, Narus JB. Prospective analysis of penile length changes after radical prostatectomy. BJU Int. 2014;113(5b):E131-6.

19. Parekh A, Chen M-H, Hoffman KE, Choueiri TK, Hu JC, Bennett CL, et al. Reduced Penile Size and Treatment Regret in Men With Recurrent Prostate Cancer After Surgery, Radiotherapy Plus Androgen Deprivation, or Radiotherapy Alone. Urology. 2013;81(1):130-5.

20. Carlsson S, Nilsson AE, Johansson E, Nyberg T, Akre O, Steineck G. Self-perceived penile shortening after radical prostatectomy. Int J Impot Res. 2012;24(5):179-84.

21. Vasconcelos JS, Figueiredo RT, Nascimento FL, Damiao R, da Silva EA. The natural history of penile length after radical prostatectomy: a long-term prospective study. Urology. 2012;80(6):1293-6.

22. Engel JD, Sutherland DE, Williams SB, Wagner KR. Changes in Penile Length After Robot-Assisted Laparoscopic Radical Prostatectomy. Journal of Endourology. 2011;25(1):65-9.

23. Tal R, Heck M, Teloken P, Siegrist T, Nelson CJ, Mulhall JP. ORIGINAL RESEARCH—PEYRONIE'S DISEASE: Peyronie's Disease Following Radical Prostatectomy: Incidence and Predictors. The journal of sexual medicine. 2010;7(3):1254-61.
